# Binaural Beat Stimulation Enhances Cognitive Function in Alzheimer’s Disease via Temporal Lobe Activation: An sLORETA Study

**DOI:** 10.3390/biomedicines14030540

**Published:** 2026-02-27

**Authors:** Muhammad Danish Mujib, Nayab Mubashir, Ahmad Zahid Rao, Nisha Nasir, Ayesha Ikhlaq, Syeda Sehar Hussain, Fizza Zia, Ghulam Mohiuddin Asim, Ahmad O. Alokaily, Mohamed A. Almadi, Saad Ahmed Qazi, Muhammad Abul Hasan

**Affiliations:** 1 Department of Biomedical Engineering, NED University of Engineering & Technology, Karachi 75270, Pakistan; danishmujib@neduet.edu.pk (M.D.M.); nm.nayab@gmail.com (N.M.); nishakhi2002@gmail.com (N.N.); seharhussain003@outlook.com (S.S.H.); fizzazia@neduet.edu.pk (F.Z.); 2 Neurocomputation Lab, National Center of Artificial Intelligence, NED University of Engineering & Technology, Karachi 75270, Pakistan; 3 Department of Physical Medicine and Rehabilitation, McGovern Medical School, University of Texas Health Science Center at Houston, Houston, TX 77030, USA; ahmad.z.rao@uth.tmc.edu; 4 Institute of Physics, Islamia University of Bahawalpur, Bahawalpur 63100, Pakistan; ayesha.ikhlaq@iub.edu.pk; 5 International Programs, Azteca University, Chalco de Díaz Covarrubias 56600, Mexico; 6 Department of Biomedical Technology, College of Applied Medical Sciences, King Saud University, Riyadh 12372, Saudi Arabia; 7 Department of Electrical Engineering, NED University of Engineering & Technology, Karachi 75270, Pakistan

**Keywords:** Alzheimer’s disease, binaural beats, EEG, brain networks, sLORETA, cognitive function

## Abstract

**Background**: The global prevalence of Alzheimer’s disease (AD) has reached 55.2 million. AD is characterized by progressive deterioration in cognition and working memory (WM), which are essential for attention, reasoning, and learning. These impairments are associated with pathological changes in cortical and subcortical regions. Binaural beats (BBs), a non-invasive auditory neuromodulation technique, have demonstrated cognitive enhancement effects in healthy individuals; however, their impact on WM in patients with AD remains largely unexplored. **Methods**: This study investigated the effects of BB stimulation on WM and cognitive function in the temporal lobe of patients with AD using standardized Low-Resolution Electromagnetic Tomography (sLORETA). Twenty-five patients with AD were randomly assigned to either an experimental group (*n* = 15) that received BB stimulation or a control group (*n* = 10) that received standard auditory stimulation. EEG recordings were obtained before and after the intervention. **Results**: Paired *t*-tests conducted on timeframe and frequency-wise sLORETA images revealed significant increases (*p* < 0.05) in theta, alpha1, and alpha2 frequency bands in the experimental group. Activated regions included the inferior, middle, superior, and transverse temporal gyri; Brodmann areas (BA) 20, 21, 22, 40, and 42; as well as networks associated with working memory and cognition. **Conclusions**: These findings suggest that BB stimulation induces temporal lobe activation, thereby enhancing working memory and cognitive function in patients with AD.

## 1. Introduction

Alzheimer’s disease (AD) is a neurological disorder that features progressive impairment of memory, cognitive abilities, and brain network integrity due to neurodegeneration. It is a major cause of dementia and is rapidly becoming one of the most expensive and burdensome diseases of this century [1]. A report by The Lancet Public Health reveals that the prevalence of AD has reached approximately 55.2 million worldwide, affecting individuals aged 65 years and above [2]. This number is estimated to rise to 78 million by 2030 and 139 million by 2050 [2]. Some researchers portray AD as a decline in the complexity of brain activity and neural dynamics [3]. However, others describe AD as a deterioration associated with localized brain changes, such as Medial Temporal Lobe (MTL) atrophy [4,5], as well as disruption of the brain networks, including the Default Mode Network (DMN) [6], Salience Network (SN) [7], Central Executive Network (CEN) [7], semantic memory network (SM) [8], Sensorimotor Network (SMN) [9], and other neural networks [10]. AD is also characterized by a progressive decline in working memory (WM), which is attributed to degeneration of the MTL, including the hippocampus, a region responsible for encoding and retrieving episodic memories [11].

WM is a core executive function that enables the temporary storage and manipulation of information. It plays a central role in higher-order cognitive–linguistic (CL) operations such as reasoning, verbal fluency, and sentence comprehension. Empirical studies have shown that WM is closely integrated with language-related processes, particularly those involving lexical-semantic access and narrative organization processes [12]. These CL functions are supported by distributed cortical networks, with critical contributions from the temporal cortex, especially the superior and middle temporal gyri, which are involved in phonological decoding, semantic integration, and verbal WM [13]. Although language processing and WM have traditionally been associated with left-lateralized temporal areas [14], recent neuroimaging studies indicate that the right temporal lobe also plays a significant role in supporting these processes, including improved verbal WM, sentence comprehension during language-based tasks [15], and enhanced attentional performance and temporal processing, particularly under high-demand perceptual conditions [16]. Thus, these findings support the perspective that bilateral, or even right-lateralization, contributes to CL and attentional processing.

The assessment techniques used in previous studies to diagnose AD severity and assess cognitive characteristics include the Mini-Mental State Examination (MMSE) [17,18,19,20]. The MMSE assesses cognitive abilities, including orientation, attention, registration, recall, calculation, language, and visual construction [21,22]. Moreover, this test is categorized into different levels of cognitive impairment, with a maximum score of 30 representing normal. Scores of 29–26 represent questionable impairment, 25–21 indicate mild impairment, 20–11 suggest moderate impairment, and scores below 10 signify severe impairment [19,23,24,25]. While such assessments are crucial for monitoring disease progression, available pharmacological treatments for AD exist and may cause side effects like headaches, diarrhea, nausea, allergies, and amyloid-related imaging abnormalities [26]; however, no curative treatment has yet been discovered [27]. To overcome this, neuromodulation techniques, such as electrical stimulation (e.g., Transcranial Direct Current Stimulation [27]) and magnetic stimulation (e.g., repetitive Transcranial Magnetic Stimulation [28]), are employed. These neuromodulation techniques have shown promising effects. In addition to electrical and magnetic neuromodulation, acoustic neuromodulation, such as gamma sensory stimulation and binaural beat (BB) stimulation, can be used to provide non-pharmacological, non-invasive treatment [28]. One of the promising approaches for cognitive enhancement in AD is the gamma sensory stimulation as highlighted by various recent studies. A systematic review of cognitive and neural effects of auditory gamma stimulation across healthy and cognitively impaired populations revealed a notable individual responses variability [29]. Other studies reported improvements in cognitive performance in mild AD patients following long-term 40 Hz auditory stimulation and reviewed the acoustic stimulation techniques emphasizing beneficial effects on memory, attention and as therapeutic target [30,31,32]. While gamma sensory stimulation primarily targets neural entrainment at a single high-frequency band, BB stimulation offers flexibility to modulate multiple frequency bands associated with working memory and attention, making it particularly suitable for examining complex cognitive functions in AD patients. BB is produced when two sound waves of slightly different frequencies are presented to each ear separately, and the difference between the two is perceived as a third tone by the brain [33]. Unlike invasive therapies, which may entail irritation, tissue and nerve trauma, memory disturbance, and infections [34,35], BB represents a safe and convenient intervention without adverse effects.

To examine the progression of neurological changes, various neuroimaging technologies have been widely utilized, including Positron Emission Tomography (PET) [36], Magnetic Resonance Imaging (MRI) [37], Functional Magnetic Resonance Imaging (fMRI) [38], and Computed Tomography (CT) [39], which have contributed significantly to revealing both anatomical and functional brain alterations. In parallel, electroencephalography (EEG) has also been instrumental in detecting abnormalities in cortical oscillatory activity, particularly through frequency-specific patterns [40,41]. fMRI and EEG are frequently used to investigate the neural effects of BB stimulation. While fMRI measures neural activity indirectly through Blood Oxygen Level-Dependent (BOLD) signals, EEG provides direct recordings with high temporal but lower spatial resolution [42]. To enhance EEG’s spatial accuracy, standardized Low-Resolution Electromagnetic Tomography (sLORETA) is used, enabling improved localization of brain activity.

sLORETA provides spatially accurate estimates of electrical activity in the brain by integrating EEG data and serves as a non-invasive method for localizing dysfunction across cortical regions with enhanced precision. Additionally, sLORETA enables three-dimensional (3D) brain mapping, a feature that provides deeper insights into the neural dynamics and network modulation induced by BB stimulation [43,44]. Understanding the neural mechanisms and the impact of targeted neuromodulation approaches requires understanding the relationship between temporal lobe dynamics and WM in AD patients. The objective of this study is to explore the impact of BB stimulation on WM and cognitive functions in AD patients, focusing on temporal lobe activation through sLORETA [45].

## 2. Methodology

### 2.1. Participants

The study comprised participants from Dar-ul-Sukun Karachi, Pakistan and Gills Shelter Center Karachi, Pakistan. The administrative authorities of both institutions provided informed consent prior to the trial. Each facility’s designated supervisory personnel were thoroughly briefed on the study protocol and granted the opportunity to review and monitor all relevant procedures both pre- and post-treatment. This process aligned fully with established ethical standards, emphasizing transparency and mutual accountability. The study was intentionally designed to foster collaborative engagement, uphold participant safety, and maintain the dignity of all stakeholders throughout its execution. The study was conducted with approval from the Research Ethics Committee, NED University of Engineering & Technology, Karachi, Pakistan on 20 November 2020 (Protocol Code: ASRB/877).

**Figure 1:**
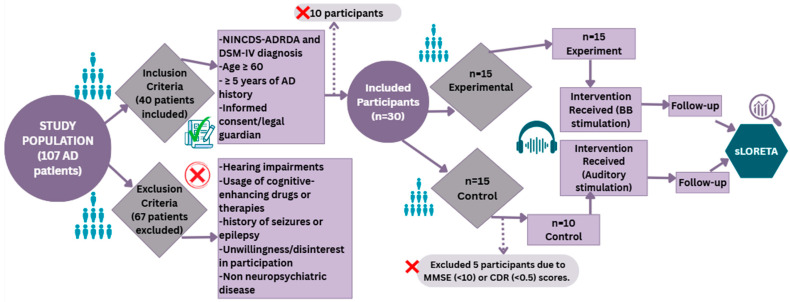
Flow diagram of participant screening, randomization, allocation, and follow-up in the study.

Initially a total of 107 random AD participants aged between 60 and 90 were screened for potential inclusion criteria as shown in Figure 1. The administration at both centers along with our research team thoroughly explained the experimental procedures to all the participants. The criteria for inclusion comprises five parameters such as evaluation of AD using the National Institute of Neurological and Communicative Disorders and Stroke–Alzheimer’s Disease and Related Disorders Association (NINCDS-ADRDA) criteria [46] and The Diagnostic and Statistical Manual of Mental Disorders, Fourth Edition (DSM-IV) [20], age of 60 years or older, at least 5 years AD history, Mini-Mental State Examination (MMSE) scores between 10 and 24 or Clinical Dementia Rating (CDR) scores between 1 and 2, and lastly, be able to provide informed consent, or obtain consent from a legal guardian in cases where participants are unable to provide it. The MMSE scores were taken using the same questionnaire by Folstein et al. [17].

The exclusion criteria encompassed the presence of other neurological or psychiatric conditions, pronounced hearing impairments that might interfere with auditory stimulation, the use of cognitive-enhancing drugs or therapies likely to impact cognitive performance, a history of seizures or epilepsy, and unwillingness or disinterest in taking part in the study. On the basis of disinterest in participating, having an AD history of less than five years, or the presence of other neurological disorders, 67 patients out of 107 were excluded from the study. The remaining 40 patients were then evaluated using NINCDS-ADRDA criteria and the DSM-IV, as a result of which only 30 patients were included, and out of which 5 were further excluded based on their MMSE (<10) or CDR (<0.5) scores. Eventually, 25 patients were selected to participate in the study.

### 2.2. Interim Analysis

This selection involved the random allocation of 30 participants to the experimental (*n* = 15) and control groups (*n* = 15) before the administration of the MMSE for unbiased distribution to assess baseline cognitive function. For significant comparisons and validity of outcomes, the criteria for inclusion/exclusion were predefined, ensuring all participants met the cognitive baseline. The allocation process of participants to either experimental or control group was carried out using a modified minimization approach. To determine the sample size of the study, an interim analysis on the initial six participants in each group (12 total) was conducted, yielding an increased effect size of *d* = 1.72 and statistical power of SP = 90% for significant changes in MMSE scores. The interim analysis revealed the effectiveness of BB intervention highlighting the significant improvements by experimental group in mean MMSE scores in pre and post states (pre-MMSE mean score = 11.57 ± 3.25, post-MMSE mean score = 17.10 ± 3.18). Therefore, on this basis, a maximum sample size of 11 participants per group, making a total of 22, was calculated to achieve *p* < 0.025 and SP = 80%, ensuring a balance between precision and feasibility. This approach offers adaptive refinement of the sample size along with optimizing resource utilization while maintaining high statistical rigor. The robustness and reliability of the findings were further enhanced in order to effectively mitigate the potential for false positives by employing a stricter significance threshold of *p* < 0.025 instead of the conventional *p* < 0.05. The control group experienced a lack of five participants (*n* = 10) since they failed to meet the minimum MMSE qualification criteria (MMSE (<10) or CDR (<0.5) scores) which were mandatory for inclusion in the study and were thus excluded thereafter. Although a slight unequal sample size appeared between the two groups due to the exclusion, the study design still maintained its robustness because of the randomized allocation process and high statistical power for the sample size. Thus, to ensure valid and generalizable findings, strict adherence to the inclusion/exclusion criteria was enforced to preserve the integrity of the analysis.

The 25 recruited AD participants comprised 16 males (71.19 ± 8.28 years) and 9 females (67.78 ± 9.40 years). These patients were randomly categorized into two groups based on the type of stimulation they received. The experimental group (*n* = 15) were administered 10 Hz alpha binaural beats whereas the control group (*n* = 10) received a standard auditory tone. The administration and the participants in both centers were blinded to the group assignments to prevent any bias in their responses; however, the researchers were aware of this allocation.

### 2.3. Experimental Protocols

**Figure 2:**
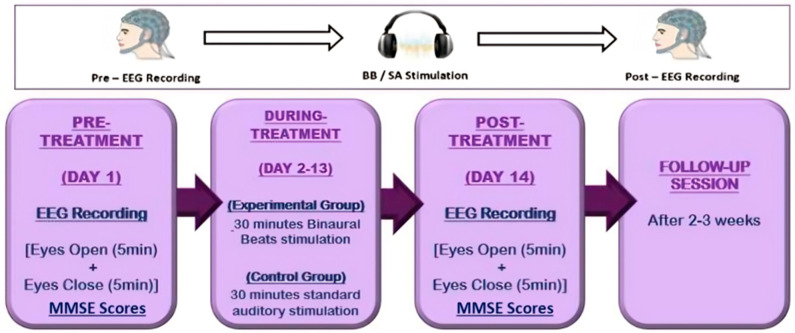
Experimental protocol.

The designed experimental protocol spanned 14 days and included pre- and post-neurological and psychometric assessments, as shown in Figure 2. On Day 1 (pre-treatment) and Day 14 (post-treatment), psychometric assessments were conducted using the MMSE. For the neurological evaluation, AD patients were asked to sit comfortably, and 38 scalp electrodes were placed according to the 10–20 system. EEG recordings were obtained using an EEG device, beginning with a 5 min eye-closed (EC) session followed by a 5 min eye-open (EO) session. From days 2 to 13 (during-treatment), the experimental group received 30 min BB stimulation sessions, while the control group received 30 min standard auditory stimulation sessions. A follow-up session consisting only of a psychometric assessment was conducted 2–3 weeks after treatment.

### 2.4. Generation of BB/Standard Auditory Tone Stimulation

Both the BB and standard auditory stimulation tones were created using Adobe Audition v3.0 (Adobe Systems Inc., San Jose, CA, USA) [47]. The stimuli were delivered at least 50 dB above each participant’s hearing threshold, determined through standardized pure-tone audiometry [19]. Prior to each session, the trainer determined the patients’ hearing threshold in a soundproof room by initiating stimulation at a starting intensity of 45 dB HL with gradual increase until participants reported clear audibility and comfort. The threshold was recorded when the patient could comfortably detect the sound in half of the trials. For understanding, the sound will be produced at 60 dB, if the given criteria for 400 Hz for a particular subject was 15 dB (45 + 15). The final stimulus intensity was then carefully calibrated in relation to each participant’s hearing threshold to guarantee a constant and comfortable loud perceived level. Based on participant feedback, slight modifications were made to ensure comfort throughout the session. The auditory evaluation was carried out on Day 1 to make sure the auditory stimuli received through SPACE stereo headphones were comfortable, easily detectable, and catered to each participant’s individual aural sensitivity.

The experimental group received 400 Hz in the left ear and 410 Hz in the right ear, generating BB stimulation at 10 Hz, whereas the control group received 400 Hz in both ears. The control condition was designed to match the experimental protocol in terms of acoustic exposure, listening duration, calibration procedure, and equipment while eliminating the interaural frequency difference required in generating a BB percept. This allowed isolation of BB specific neural effects from general auditory stimulation. This control condition was designed as an active auditory control rather than a placebo. Participants received real auditory stimulation matched to the experimental condition in structure, duration, and delivery, but without the interaural frequency difference required to generate a BB. This enabled differentiation between neural effects specific to BB processing and those related to general auditory exposure or listening engagement, thereby serving as a physiologically relevant comparison and strengthening the study’s internal validity. Participants in both groups underwent identical procedures and were not informed about the acoustic distinctions between conditions, minimizing expectancy bias and maintaining comparable listening engagement.

Various human EEG and cognitive studies have consistently chosen 400 Hz to investigate BBS which has been shown to produce a stable binaural percept without discomfort [48,49]. The 10 Hz BB was chosen based on the evidence from previous studies that α-BB significantly improves mood, working memory, attention, emotional regulation and EEG coherence [49,50]. Moreover, multiple prior studies involving BB frequencies have been evaluated targeting beta and γ frequencies; however, more reliable and reproducible effects of cognitive performance, memory, and mood related outcomes in healthy participants were achieved using α-frequency stimulation. For this reason, the present study directly focused on α-band (10 Hz) stimulation [33,51,52]. In comparison, the control group received a single 400 Hz pure tone to match auditory stimulation while ensuring isolation of BBS’s specific neural effects. To preserve comparable perceived loudness and ensure that observed effects were attributable to BBS rather than intensity differences, the identical calibration procedure, headphones, and acoustic conditions were employed for both groups. The use of an identical carrier tone in both ears ensured comparable perceived loudness and auditory experience while avoiding additional neuromodulatory influences that may arise from alternative sham paradigms such as monaural beats or different binaural frequencies. Throughout the intervention, participant compliance and tolerability were tracked, all sessions were conducted under observation, and no withdrawals or negative effects were noted.

### 2.5. EEG Recording

Brain activity was recorded using the Mitsar-NVX EEG device (Mitsar Co. Ltd., St. Petersburg, Russia), which captured signals from 38 scalp locations covering frontal, temporal, central, parietal, and occipital regions according to the international 10–20 system, with two reference electrodes [53]. EEG recordings were obtained on Day 1 (pre-treatment) and Day 14 (post-treatment) and sampled at 500 Hz. Data acquisition was conducted in a quiet, well-ventilated room to minimize noise, and saline solution was used as an electrolyte gel to ensure good electrode conductivity.

### 2.6. Data Filtration and Processing

Once the raw EEG data were acquired, the signals were filtered and artifacts were removed using detrending and DC offset removal in MATLAB R2019a (MathWorks, Natick, MA, USA). A 5th-order Butterworth bandpass IIR filter (1–45 Hz) was applied to eliminate power-line artifacts. In addition, ocular and muscular artifacts, including eye blinks and EMG activity, were removed by visual inspection. The filtered EEG data were then processed in MATLAB using the EEGLab toolbox to segment the data into 2 s epochs at a sampling rate of 500 Hz for both the experimental and control groups. Subsequently, source localization techniques were applied to identify deep cortical structures and brain networks relevant to the study.

### 2.7. Source Localization Using sLORETA

Source localization was performed using sLORETA 2008 software, which estimates intracortical electrical activity by solving the inverse problem with a realistic Montreal Neurological Institute (MNI) head model. This software computed the current source density (CSD) of EEG epochs using a 3D MNI model. The 3D MNI space provides anatomical locations of Brodmann areas (BAs), voxel-based mapping and eight EEG frequency bands for neural activity analysis, including delta (0.1–4 Hz), theta (4–8 Hz), alpha1 (8–12 Hz), alpha2 (12–13 Hz), beta1 (13–16 Hz), beta2 (16–20 Hz), beta3 (20–30 Hz) and gamma (30–80 Hz). The estimated epochs were then analyzed in sLORETA to assess deep cortical structures and functional networks in AD patients undergoing BB stimulation. The results were visualized as tomographic maps of brain activity across three orthogonal planes (axial, sagittal, and coronal) to provide a comprehensive representation of cortical activation [54]. Axial views offered a top-down perspective, sagittal views showed lateral sections, and coronal views presented a frontal section of the brain.

### 2.8. Statistical Analysis

To determine the effect size and to assess whether the effects of training are significant, Cohen’s method was applied, which ensured that significant neurological changes at post-treatment (Day 14) were not due to false positives [47]. Effect sizes were calculated for changes in MMSE scores and EEG relative power by subtracting the group means and dividing the result by the pooled standard deviation as shown in Equation (1) [55]. An effect size greater than 0.8 was interpreted as large, whereas values between 0.4 and 0.8 were considered moderate, and those below 0.4 were considered small.

(1) $$cohen\;d=\frac{{\bar{x}}_{1}-{\bar{x}}_{2}}{\sqrt{\frac{{SD}_{1}\left(n_{1}-1\right)+{SD}_{2}\left(n_{2}-1\right)}{n_{1}+n_{2}-2}}}\;$$

where ${\bar{x}}_{1}$ and ${\bar{x}}_{2}$ are mean values, ${SD}_{1}$ and ${SD}_{2}$ are the standard deviations, and $n_{1}$ and $n_{2}$ are the sample sizes of the two groups.

Statistical analysis in sLORETA was performed using timeframe and frequency-wise normalization to ensure sensitivity and relevance of the source localization results. sLORETA functional images were computed separately for each subject and condition across eight EEG frequency bands. To enhance spatial specificity, the images were normalized on a frequency-band basis using the timeframe-wise normalization option in the software. This normalization method scaled the average power across all voxels to unity for each frequency band within each subject. This analysis focused on spatial distribution differences in cortical activity between conditions, minimizing the confounding influence of inter-individual variability in global EEG signal strength [56]. Statistically significant changes in cortical activation were identified by comparing pre- and post-treatment conditions using a paired *t*-test (*p* < 0.05), with the F-ratio evaluated using a log-transformed statistic. Correction for multiple comparisons was performed using a nonparametric single-threshold test based on a randomization and permutation procedure. If any voxel value (t-value), established by 5000 randomizations, exceeded the critical threshold, the omnibus null hypothesis, which states that there was no activation anywhere in the brain, was rejected [57,58].

## 3. Results

The 25 recruited AD participants included were 16 males (age: 71.19 ± 8.28 years) and 9 females (age: 67.78 ± 9.40 years). All patients completed the intervention, and there was no loss to follow-up.

### 3.1. Results of Cognitive Function Assessment

MMSE cognitive assessment revealed significant changes. Mean MMSE scores and standard deviations (SDs) for the experimental and control groups in both pre-treatment and post-treatment conditions are shown in Table 1. Mean values were calculated for all MMSE components, including orientation, registration, attention, recall, language, and copying. Bold values indicate statistically significant changes following administration of either BB or auditory stimulation, depending on the group.

At the end of the 14-day protocol, the experimental group showed significant improvement in orientation (*p* < 0.001), recall (*p* = 0.034), and language (*p* = 0.003). However, MMSE components, including attention, registration, and copying, did not show significant improvement following BB stimulation. In contrast, the control group did not show a significant change in any MMSE component following auditory stimulation, except for language (*p* < 0.001). Similarly, follow-up MMSE scores showed significant increase in the experimental group relative to pre-treatment values (*p* <0.001, t = 7.27), whereas no significant improvement was observed in the control group (*p* = 0.42). A significant overall difference between experimental and control groups was observed with variable MMSE scores (*p* = 0.016), and an effect size (η^2^ = 0.063) indicating that 6.3% of the variance was explained by group difference. MMSE scores also differed significantly across sessions of pre, post and follow-up (*p* = 0.024) and effect size (η^2^ = 0.079), accounting for 7.9% of the variance. However, no significant interaction was found between group and session (*p* = 0.102), suggesting that the pattern of change over time was comparable between the two groups.

### 3.2. sLORETA-Based Cortical Activation in WM and Cogntive Functions

**Figure 3:**
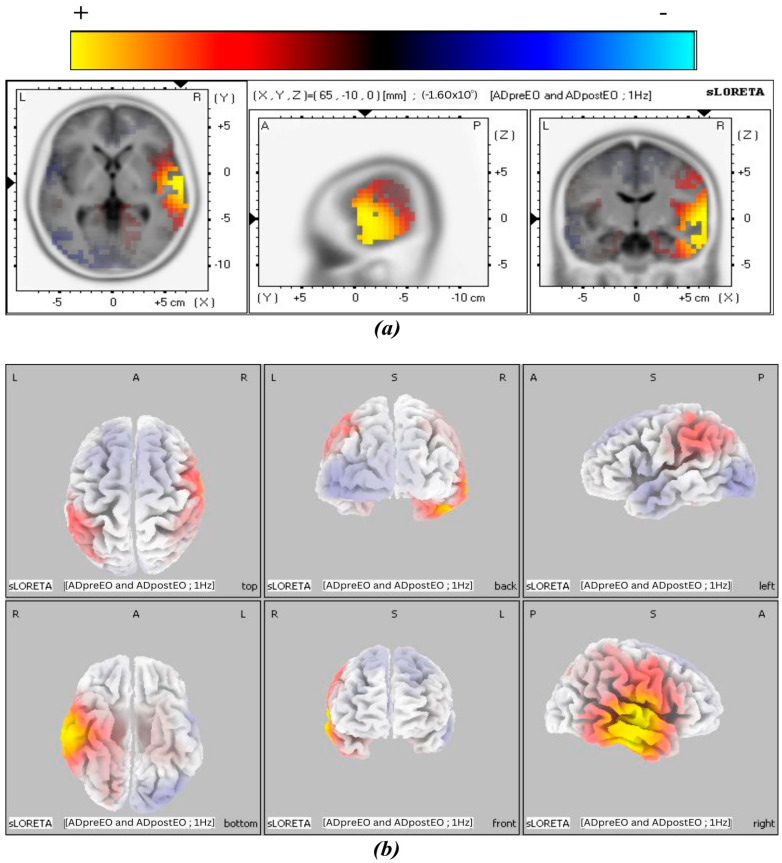
MNI slices illustrating the effect of BB stimulation in the post-treatment of the experimental group for the theta band. Panel (**a**) shows slice views of cortical current density, and panel (**b**) shows a 3D brain cortex from six viewpoints, with the corresponding color scale bar.

**Figure 4:**
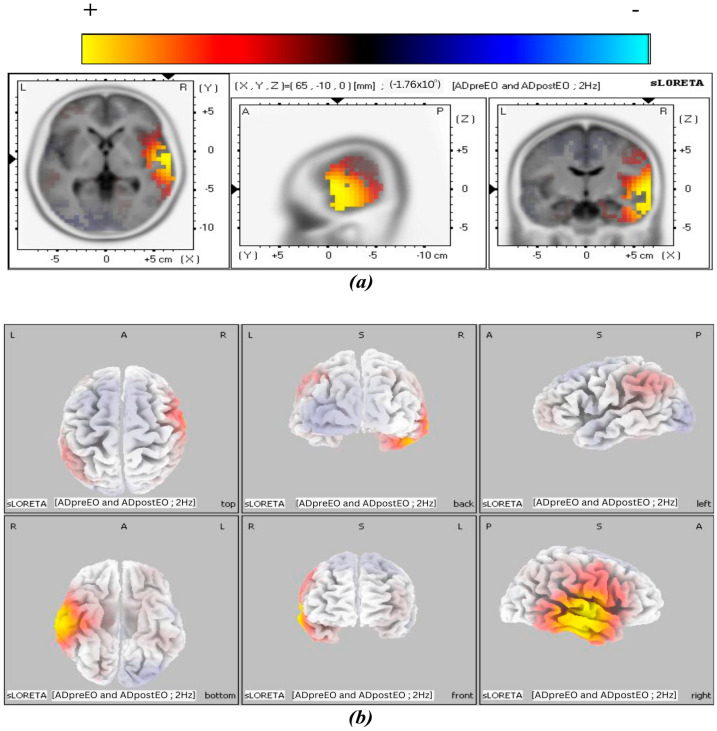
MNI slices illustrate the effect of BB stimulation in the post-treatment condition of the experimental group for the alpha1 band. Panel (**a**) shows slice views of cortical current density, and panel (**b**) shows a 3D brain cortex from six viewpoints, with the corresponding color scale bar.

**Figure 5:**
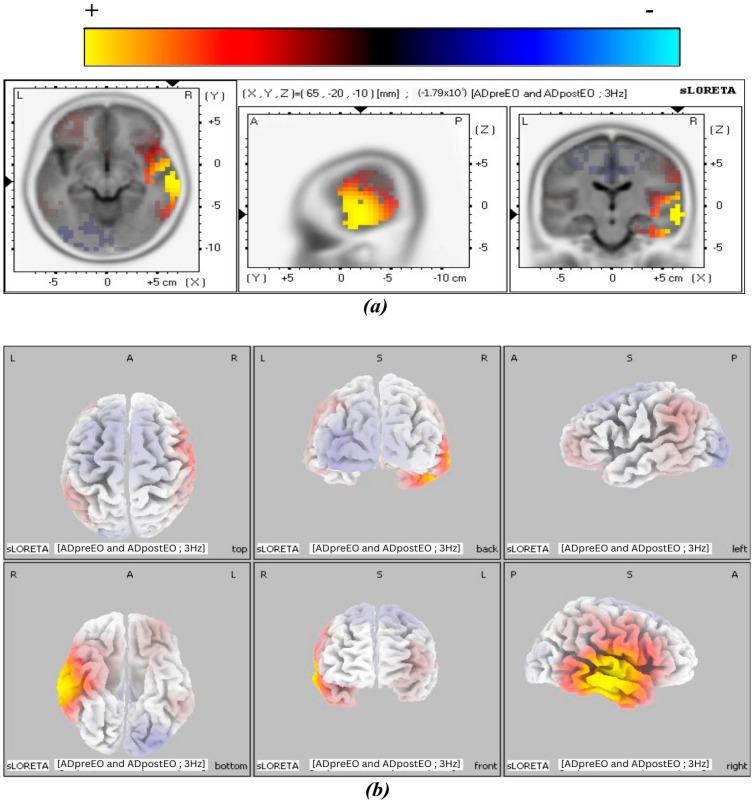
MNI slices illustrating the effect of BB stimulation in the post-treatment condition of the experimental group for the alpha2 band. Panel (**a**) shows a slice view of cortical current density, and panel (**b**) shows a 3D brain cortex from six viewpoints, with the corresponding color scale bar.

**Figure 6:**
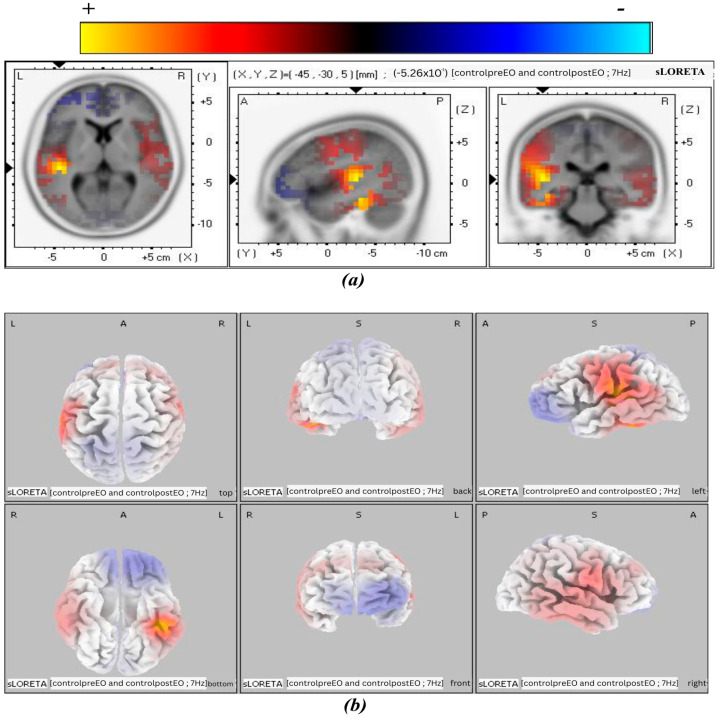
MNI slices illustrating the effect of auditory stimulation in the post-treatment condition of the control group for the beta3 band. Panel (**a**) shows a slice view of cortical current density, and panel (**b**) shows a 3D brain cortex from six viewpoints, with the corresponding color scale bar.

The 3D tomographic reconstructions were carried out using sLORETA across all frequency bands in both the experimental and control groups. Significant activation was observed in the theta, alpha1, and alpha2 frequency bands in the post-treatment condition of the experimental group, whereas only the beta3 band in the control group showed significant activation in the control group. The spatial distribution of neuronal activity in Figure 3, Figure 4, Figure 5 and Figure 6 is shown using color-coded statistical parametric maps, with warmer colors indicating higher cortical current density and cooler colors indicating lower density.

Figure 3 illustrates the slice views of the estimated cortical current density distribution in the theta frequency band of the experimental group in the post-treatment condition. Increased activation is observed in the tomographic views, particularly in the right temporal cortex. The strongest signals are localized to lateral and temporal regions. The 3D surface projections confirm that this activation is predominantly right-lateralized and localized to the middle and superior temporal gyri. This pattern suggests that theta-band modulation engages specific temporal regions following BB stimulation, without widespread cortical involvement.

Figure 4 shows results of cortical current density for the alpha1 band. Compared with the theta band, the spatial extent of activation appears more pronounced in the alpha1 band. The maps reveal continued right-lateralized activation, with additional involvement of surrounding temporal regions. The middle temporal gyrus (MTG) exhibits stronger and more spatially extended increases in current density. The 3D views highlight greater surface expression, particularly across the inferior and posterior temporal cortex. These findings suggest a broader cortical response in the alpha1 frequency band, centered in the right hemisphere.

Figure 5 presents the alpha2 band results of cortical current density, showing greater magnitude and spatial extent of activation. Panel (a) depicts slice views demonstrating the distribution of cortical current density, while panel (b) provides a 3D cortical representation of the brain visualized from six viewpoints. The most prominent activation cluster is in the right MTG, with expanded coverage into inferior temporal regions. Greater signal intensity and broader distribution along the lateral and basal aspects of the temporal lobe are evident in both tomographic and surface views. Signal strength and spatial extent increased across the alpha frequency bands. This pattern shows a progressive cortical response at higher alpha frequencies, localized primarily to the temporal cortex.

Figure 6 presents the cortical activation pattern observed in the control group for the beta3 frequency band at the post-treatment period. Among all frequency bands analyzed, beta3 was the only band that showed a measurable increase in cortical current density. Activation is localized to the left superior temporal gyrus (STG), as evidenced by the axial, sagittal, and coronal sLORETA slice views. The increase in current density appears spatially restricted, with no additional suprathreshold clusters observed in other cortical regions.

### 3.3. Quantitative Assessment of Cortical Activation Underlying Cognitive Performance

Statistically significant changes (*p* < 0.05) in deep cortical structures were observed in the theta, alpha1, and alpha2 frequency bands in the experimental group and are summarized in Table 2. The table highlights distinct cortical regions, percentages of activated voxels, and corresponding MNI coordinates, reflecting changes across multiple brain networks and BAs in AD patients after treatment. Notably, these activations were predominantly localized to the right hemisphere post-treatment in the experimental group for these frequency bands.

The remaining frequency bands, including beta1, beta2, beta3, and gamma, did not show significant activation following BB stimulation. Table 2 supports the visual patterns observed in the sLORETA maps through voxel-wise quantification. In the theta band, activation was observed in 2% of voxels within both the MTG (BA 21) and the STG (BA 22). In the alpha1 band, voxel engagement increased to 20% in BA 21 and 12% in BA 22, with additional activation in BA 20 (5%) and BA 42 (8%). The alpha2 band demonstrated the highest involvement, with 25% of voxels active in BA 21, along with additional activation in BA 20 (6%), BA 22 (12%), and BA 42 (15%).

These findings indicate a frequency-specific increase in activation magnitude and spatial distribution, particularly within right temporal cortical regions implicated in auditory and associative processing. In contrast, the control group showed significant activation only in the beta3 band in BA 41, which is associated with the auditory network. This activation showed a peak current density of 0.53, with voxel engagement of 1.82%. The activation was limited to the left hemisphere and was not accompanied by significant changes in any other frequency bands examined in the control group. No additional voxel clusters exceeded threshold criteria across theta, alpha1, alpha2, or other frequency bands, and no bilateral or diffuse patterns were detected in the post-treatment condition for this group.

## 4. Discussion

The study aimed to assess neurological and psychometric variations in the temporal lobe induced by BB stimulation in AD patients and to explore its impact on WM and CL functions using sLORETA. The control condition employed a matched carrier tone without interaural frequency disparity to isolate BB specific effects rather than introduce another entrainment stimulus, ensuring that observed neural and cognitive changes were attributable to beat related mechanisms rather than general auditory stimulation. The results show significant improvements in cognitive functions at both psychometric and neurological levels following stimulation. Increased neurological changes were observed in the theta, alpha1, and alpha2 frequency bands in the experimental group, consistent with the psychometric improvements measured in MMSE scores. These findings suggest a mechanism through which BB stimulation enhances WM and cognitive functioning in AD patients.

Changes in MMSE scores in the experimental group indicate a clear effect of BB stimulation. Improvements in specific MMSE components following the intervention highlight the potential of BB stimulation to enhance orientation, registration, and language functions. These improvements may help mitigate cognitive decline, behavioral changes, memory loss, and communication difficulties associated with AD. These findings are consistent with previous studies conducted on non-AD populations that have explored the cognitive benefits of BB interventions in dementia-related conditions [59,60]. The group-level analysis further supported these findings, showing a significant main effect of group and session on MMSE scores, with small-to-moderate effect sizes. This indicates that cognitive performance differed between experimental and control groups and varied across assessment time points. These results imply that while BB stimulation may be associated with overall cognitive differences, its temporal effects should be interpreted cautiously.

The observed neural changes showed significant increase in neural activity in the theta, alpha1, and alpha2 frequency bands in the post-treatment condition of the experimental group, whereas the beta1, beta2, beta3, and gamma bands did not show a significant change. A study by Moretti reported that elevated theta activity is associated with preservation of attention and memory, suggesting a role in compensatory mechanisms during preclinical AD [61,62]. Moretti also reported increased alpha1 and alpha2 power, which were associated with greater hippocampal perfusion and reduced cortical atrophy, reflecting the functional integrity of memory and attention circuits in patients with MCI [61]. These findings may suggest that BB intervention effectively modulates neural activity in brain regions associated with memory, language processing, auditory perception, and sensorimotor functions, thereby influencing brain networks crucial for cognitive function in AD [63,64,65].

Theta-band activation was predominantly observed in BA21 and BA22 (MTG and STG), which are integral to WM and auditory processing. Mummery et al. suggested that these BAs are involved in semantic memory processing, visual perception, and language processing [66]. Theta-band activity has also been widely associated with hippocampal function, attention regulation, and episodic memory formation, all of which are disrupted in AD [67]. The significant right-hemisphere activation observed in the MTG further supports the role of theta-band activity in memory consolidation and cognitive processing, which may be particularly relevant for patients with language and semantic retrieval difficulties. This interpretation is consistent with previous research demonstrating the involvement of theta band in cognitive processing, particularly WM consolidation [68,69]. Moreover, Trammell et al. reported that increased task-related theta/alpha ratios (TARs) are associated with enhanced cognitive performance, including short-term memory and reasoning, in both young and aging adults, with theta activity contributing directly during task execution [70]. Left-hemispheric temporal regions are classically associated with language functions; however, the contribution of right temporal cortex in higher-order language, attentional control, and verbal working memory processes, particularly under cognitively demanding conditions and in aging or neurodegenerative populations has been indicated by growing neuroimaging evidences [71,72]. BA21 and 22 showing right-lateralized activation has been linked with compensatory neural recruitment supporting memory-related language processing, semantic integration and auditory attention. Therefore, reflecting adaptive or compensatory reorganization rather than contradiction of established left-lateralized language models, especially in AD populations where bilateral and right-hemisphere engagement, is commonly reported [73].

Results in the alpha1 band showed a significant increase in activation across BA6, BA20, BA21, BA22, and BA40. The alpha band is closely linked to cognitive processing efficiency, attentional control, and WM maintenance. Increased alpha1 activity in BA20 and BA21 aligns with previous studies demonstrating that alpha-frequency BB stimulation improves sustained attention and task performance [74]. Findings reported by Babiloni et al. show that higher resting-state alpha1/alpha2 activity positively correlates with greater cognitive stability, particularly in memory and attention, in patients with MCI [10].

The BA40 enhancement in the DMN within the postcentral gyrus (POG) suggests that BB stimulation may improve attentional networks and mitigate cognitive decline associated with AD, thereby facilitating improved concentration and task performance. This interpretation is consistent with findings reported by Abul Hasan et al., who found that increased parietal activity is associated with improvements in WM performance and visual attention [75].

Moreover, BA6 is associated with precentral gyrus (PRG), located within the Premotor Cortex, which plays a crucial role in executive functioning [76]. Stein et al. reported that increased activity in these cortical regions may support motor planning and coordination, which may benefit AD patients by supporting daily activities and cognitive functions involving motor control, potentially reducing reaction time [66,77].

In addition, BA42, part of the primary auditory cortex, plays a significant role in phonological processing, including higher-order auditory perception, auditory WM, and speech processing. In this study, increased activation in BA42 (MTG) and BA22 (STG) was observed in response to BB stimulation in the theta band. This finding aligns with prior evidence indicating that activation in these BAs reflects their functional involvement not only in sound detection but also in phoneme perception and language processing [78].

However, in the control group, time–frequency and independent statistical analyses (*p* < 0.05) revealed a significant increase in brain activity only in the beta3 band, specifically localized to the STG corresponding to BA41. Although the control group exhibited a significant increase in beta3 band activity following auditory tone stimulation, this effect likely reflects a generalized state of cortical arousal or sensory responsiveness rather than targeted cognitive enhancement. In contrast, BB stimulation produced broader and more functionally relevant frequency changes, suggesting a stronger association with memory and cognitive processing [59,79].

Our study findings are consistent with prior research suggesting that increased temporal and parietal activity is associated with improved executive functions, such as WM and visual attention, in AD patients following BB stimulation. The observed brain activation patterns and neural networks in the experimental group showed that BB stimulation had a positive impact not only on memory but also on other cognitive and linguistic functions.

Despite the potential of BB, we acknowledge several limitations of the present study including the absence of neurological assessment during the follow-up sessions. While psychometric assessments provide important behavioral evidence of memory improvement, EEG markers would allow direct evaluation of long-term neural changes induced by BB. Moreover, the study is a single blind investigation and the sample size in this study is limited; studies with larger participant numbers could provide more robust and generalizable insights. Another limitation includes the short duration of intervention duration, restricting the assessment of long-term effects. Therefore, the present study should be considered preliminary and hypothesis-generating.

Although preliminary, the observed cognitive and neural improvements suggest that BB stimulation may have practical benefits in clinical settings for patients with AD. Short sessions of BB could be integrated into cognitive rehabilitation programs to support memory, attention, and language, potentially helping patients maintain functional independence. Given its non-invasive and low-cost nature, BB may complement existing therapies. However, due to the small sample, short intervention, and the use of estimated neural measures via sLORETA, these findings should be interpreted cautiously, and larger clinical trials are needed to confirm efficacy, optimize protocols, and evaluate long-term outcomes.

Future studies with larger, more diverse cohorts, extended intervention periods, and fully blinded designs are warranted to confirm and extend these results. Moreover, future studies should include follow-up EEG measurements to determine whether the observed cognitive enhancements are supported by sustained neural plasticity rather than transient entrainment effects. It may leverage machine learning (ML) models to stratify patient responses and personalize BB stimulation protocols, an approach already being explored for WM enhancement [80]. Moreover, integrating complementary stimulation techniques such as Peltier-based peripheral neuromodulation [81], as well as other neuromodulation therapies including Transcranial Direct Current Stimulation (tDCS), Transcutaneous Electrical Nerve Stimulation (TENS) [75,82], and neurofeedback [83] could help establish a practical, clinic-ready framework for AD management. Overall, these findings suggest that BB stimulation represents a promising non-invasive neuromodulatory approach for enhancing cognition and WM in AD.

## 5. Conclusions

Overall, this study suggests that BB stimulation induces targeted activation in the temporal lobe and associated cortical structures in individuals with AD. The engagement of regions such as the inferior, middle, and superior temporal gyri, along with precentral and postcentral areas, may reflect modulation of neural networks underlying WM and CL processing, although sLORETA provides an estimate of cortical activity rather than direct measurement. Enhanced activation across multiple brain networks, including the SM, DMN, LN, AN, and SMN, may be associated with the role of BB stimulation in facilitating auditory–verbal integration and memory-related functions in AD. Collectively, these findings suggest the potential of BB as a non-invasive neuromodulatory intervention for supporting cognitive function in AD.

## Figures and Tables

**Table 1:** Mean MMSE scores ± SD for the experimental and control groups across pre, post-treatment, and follow-up conditions. Bold values indicate statistically significant changes following BB stimulation.

MMSE (All Parts)	Experimental (Mean ± SD)		Control (Mean ± SD)	
	Pre	Post	Pre	Post
Orientation	4.73 ± 2.54	**7.27 ± 1.57**	4.7 ± 2.05	5.2 ± 1.94
Registration	2.27 ± 0.93	2.47 ± 0.72	1.3 ± 0.78	1.2 ± 0.6
Attention	1.47 ± 1.63	2 ± 1.75	1.3 ± 1.79	1.4 ± 1.74
Recall	1.13 ± 0.81	**1.6 ± 1.14**	1.6 ± 1.02	1.6 ± 0.8
Language	3.53 ± 2.16	**4.67 ± 2.27**	2.3 ± 1.68	**3.6 ± 1.2**
Copying	0.33 ± 0.47	0.47 ± 0.5	0.2 ± 0.4	0.2 ± 0.4
Follow-up MMSE scores				
(mean ± SD)	**18.76 ± 5.04**		15.01 ± 4.11	

**Table 2:** Significant change in BAs observed in the theta, alpha1 and alpha2 frequency bands in the experimental group whereas only beta3 showed significant change in the eyes open (EO) condition for post-treatment (*p* = 0.05), while other frequency bands did not reach statistical significance.

Frequency Band	Cortical Area	Brain Networks	BAs	MNI Coordinates with Min Values	Max Activation	Voxels (%) (Post)
Experimental Group						
theta	Middle Temporal Gyrus	SM	21	(60, 15, −10)	1.60 × 10^0^	2% (right)
	Superior Temporal Gyrus	LN	22	(65, −10, 0)	1.60 × 10^0^	2% (right)
alpha1	Precentral Gyrus	SMN	6	(55, −5, 5)	1.61 × 10^0^	0% (right)
	Inferior Temporal Gyrus	SM	20	(65, −15, −25)	1.72 × 10^0^	5% (right)
	Middle Temporal Gyrus	SM	21	(65, −10, −5)	1.75 × 10^0^	20% (right)
	Superior Temporal Gyrus	LN	22	(65, −10, 0)	1.76 × 10^0^	12% (right)
	Transverse Temporal Gyrus	AN	42	(65, −10, 10)	1.69 × 10^0^	8% (right)
alpha 2	Inferior Temporal Gyrus	SM	20	(65, −20, −20)	1.78 × 10^0^	6% (right)
	Middle Temporal Gyrus	SM	21	(65, −20, −10)	1.79 × 10^0^	25%(right)
	Superior Temporal Gyrus	LN	22	(65, −20, 0)	1.76 × 10^0^	12% (right)
	Transverse Temporal Gyrus	AN	42	(65, −10, 10)	1.68 × 10^0^	15% (right)
Control Group						
beta3	Superior Temporal Gyrus	AN	41	(−45, −30, 5)	5.26 × 10^−1^	1.82% (left)

## Data Availability

The datasets generated and/or analyzed during the current study are not publicly available due to privacy and ethical concerns but are available from the corresponding author upon reasonable request.
